# What is useful research? The good, the bad, and the stable

**DOI:** 10.1186/s12940-019-0556-5

**Published:** 2020-01-07

**Authors:** David M. Ozonoff, Philippe Grandjean

**Affiliations:** 1 Department of Environmental Health, Boston University School of Public Health, Boston, MA USA; 2 Department of Environmental Medicine, University of Southern Denmark, Odense, Denmark; 3 Department of Environmental Health, Harvard T.H. Chan School of Public Health, Boston, MA USA

**Keywords:** Generalizability, Quality, Replication, Reproducibility, Scientific journals, Validity

## Abstract

A scientific journal like *Environmental Health* strives to publish research that is useful within the field covered by the journal’s scope, in this case, public health. Useful research is more likely to make a difference. However, in many, if not most cases, the usefulness of an article can be difficult to ascertain until *after* its publication. Although replication is often thought of as a requirement for research to be considered valid, this criterion is retrospective and has resulted in a tendency toward inertia in environmental health research. An alternative viewpoint is that useful work is “stable”, i.e., not likely to be soon contradicted. We present this alternative view, which still relies on science being consensual, although pointing out that it is not the same as replicability, while not in contradiction. We believe that viewing potential usefulness of research reports through the lens of stability is a valuable perspective.

## Good science as a purpose

Any scientific journal wishes to add to the general store of knowledge. For *Environmental Health,* an additional important goal is also to publish research that is useful for public health. While maximizing scientific validity is an irreducible minimum for any research journal, it does not guarantee that the outcome of a “good” article is useful. Most writing on this subject concerns efficiencies and criteria for generating new and useful research results while avoiding “research waste” [1]. In this regard, the role of journals is hard to define. Indeed, a usefulness objective depends upon what happens *after* publication, thus to some extent being out of our control. That said, because of the importance of this issue the Editors have set out to clarify our thinking about what makes published research useful.

First the obvious: properly conducted scientific research may not be useful, or worse, may potentially mislead, confuse or be erroneously interpreted. Journal editors and reviewers can mitigate such regrettable outcomes by being attentive to faulty over- or under-interpretation of properly generated data, and vice versa, ensuring that unrealistic standards don’t prevent publication of a “good” manuscript. In regard to the latter, we believe our journal should not shy away from alternative or novel interpretations that may be counter to established paradigms and have consciously adopted a precautionary orientation [2]: We believe that it is reasonable to feature risks that may seem remote at the moment because the history of environmental and occupational health is replete with instances of red flags ignored, resulting in horrific later harms that could no longer be mitigated [3, 4].

Nonetheless, it has happened that researchers publishing results at odds with vested interests have become targets of unreasonable criticism and intimidation whose aim is to suppress or throw suspicion on unwelcome research information, as in the case of lead [3, 5] and many other environmental chemicals [6]. An alternative counter strategy is generating new results favorable to a preferred view [7, 8], with the objective of casting doubt on the uncomfortable research results. Indeed, one trade association involved in supporting such science once described its activities with the slogan, “Doubt is our product” [9]. Thus, for better or for worse, many people do not separate science, whether good or bad, from its implications [10].

Further, even without nefarious reasons, it is not uncommon for newly published research to be contradicted by additional results from other scientists. Not surprisingly, the public has become all too aware of findings whose apparent import is later found to be negligible, wrong, or cast into serious doubt, legitimately or otherwise [11]. This has been damaging to the discipline and its reputation [12].

## Replication as a criterion

A principal reaction to this dilemma has been to demand that results be “replicated” before being put to use. As a result, both funding agencies [13] and journals [14] have announced their intention of emphasizing the reproducibility of research, thereby also facilitating replication [15]. On its face this sounds reasonable, but usual experimental or observational protocols are already based on internal replication. If some form of replication of a study is desired, attempts to duplicate an experimental set-up can easily produce non-identical measurements on repeated samples, and seemingly similar people in a population may yield somewhat different observations. Given an expected variability within and between studies, we need to define more precisely what is to be replicated and how it is to be judged.

That said, in most instances, it seems that what we are really asking for is *interpretive* replication (i.e., do we think two or more studies *mean* the same thing), not observational or measurement replication. Uninterpreted evidence is just raw data. The main product of scientific journals like *Environmental Health* is *interpreted* evidence. It is interpreted evidence that is actionable and likely to affect practice and policy.

## Research stability

This brings us back to the question of what kind of evidence and its accompanying interpretation is likely to be of use? The philosopher Alex Broadbent distinguishes between how results get used and the decision about *which* results are likely to be used [16]. Discussions of research translation tend to focus on the former question, while the latter is rarely discussed. Broadbent introduces a new concept into the conversation, the *stability* of the research results.

He begins by identifying which results are *not* likely to be used. Broadbent observes that if a practitioner or policy-maker thinks a result might soon be overturned she is unlikely to use it. Since continual revision is a hallmark of science, this presents a dilemma. All results are open to revision as science progresses, so what users and policy makers really want are *stable* results, ones whose meaning is unlikely to change in ways that make a potential practice or policy quickly obsolete or wrong. What are the features of a stable result?

This is a trickier problem than it first appears. As Broadbent observes it does not seem sufficient to say that a stable a result is one that is not contradicted by subsequent work, an idea closely related to replication. Failure to contradict, like lack of replication, may have many reasons, including lack of interest, lack of funding, active suppression of research in a subject, or external events like social conflict or recession. Moreover, there are many examples of clinical practice, broadly accepted as stable in the non-contradiction sense, that have not been tested for one reason or another. Contrariwise, contradictory results may also be specious or fraudulent, e.g., due to attempts to make an unwelcome result appear unstable and hence unusable [6, 9]. In sum, lack of contradiction doesn’t automatically make a result stable, nor does its presence annul the result.

One might plausibly think that the apparent *truth* of a scientific result would be sufficient to make a result stable. This is also in accordance with Naomi Oreskes’ emphasis of scientific knowledge being fundamentally consensual [10] and relies on the findings being generalizable [15]. Our journal, like most, employs conventional techniques like pre-publication peer review and editorial judgment, to maximize scientific validity of published articles; and we require Conflict of Interest declarations to maximize scientific integrity [6, 17]. Still, a result may be true but not useful, and science that isn’t true may be very useful. Broadbent’s example of the latter is the most spectacular. Newtonian physics continues to be a paragon of usefulness despite the fact that in the age of Relativity Theory we know it to be false. Examples are also prevalent in environmental health. When John Snow identified contaminated water as a source of epidemic cholera in the mid-nineteenth Century he believed a toxin was the cause, as the germ theory of disease had not yet found purchase. This lack of understanding did not stop practitioners from advocating limiting exposure to sewage-contaminated water. Nonetheless, demands for modes of action or adverse outcome pathways are often used to block the use of new evidence on environmental hazards [18].

## Criteria for stability

Broadbent’s suggestion is that a result likely to be seen as stable by practitioners and policy makers is one that (a) is not contradicted by good scientific evidence; and (b) would not *likely* be soon contradicted by further good research [16] (p. 63).

The first requirement, (a), simply says that any research that produces contradictory evidence be methodologically sound and free from bias, i.e., “good scientific evidence.” What constitutes “good” scientific evidence is a well discussed topic, of course, and not a novel requirement [1], but the stability frame puts existing quality criteria, in a different, perhaps more organized, structure, situating the evidence and its interpretation in relation to stability as a criterion for usefulness.

More novel is requirement (b), the belief that *if* further research were done it would not likely result in a contradiction. The *if* clause focuses our attention on examining instances where the indicated research has not yet been done. The criterion is therefore prospective, where the replication demand can only be used in retrospect.

This criterion could usefully be applied to inconclusive or underpowered studies that are often incorrectly labeled “negative” and interpreted to indicate “no risk” [18]. A U.S. National Research Council committee called attention to the erroneous inference that chemicals are regarded inert or safe, unless proven otherwise [19]. This “untested-chemical assumption” has resulted in exposure limits for only a small proportion of environmental chemicals, limits often later found to be much too high to adequately protect against adverse health effects [20, 21]. For example, some current limits for perfluorinated compounds in drinking water do not protect against the immunotoxic effects in children and may be up to 100-fold too high [22].

## Inertia as a consequence

Journals play an unfortunate part in the dearth of critical information on emerging contaminants, as published articles primarily address chemicals that have already been well studied [23]. This means that environmental health research suffers from an impoverishing inertia, which may in part be due to desired replications that may be superfluous or worse. The bottom line is that longstanding acceptance in the face of longstanding failure to test a proposition should not be used as a criterion of stability *or* of usefulness, although this is routinely done.

If non-contradiction, replication or truth are not reliable hallmarks of a potentially useful research result, then what is? Broadbent makes the tentative proposal that a stable interpretation is one which has a satisfactory answer to the question, “Why *this* interpretation rather than another?” Said another way, are there more likely, almost or equally as likely, or other possible explanations (including methodological error in the work in question)? Sometimes the answer is patently obvious. Such an evaluation is superfluous in instances where the outcomes have such forceful explanations that this exercise would be a waste of time, for example a construction worker falling from the staging. We only need one instance and (hopefully no repetitions) to make the case.

## Consensus and stability

Having made the argument for perspicuous interpretation, we must also issue a note of caution. It is quite common to err in the other direction by downplaying conclusions and implications. Researchers frequently choose to hedge their conclusions by repeated use of words such as ‘maybe’, ‘perhaps’, ‘in theory’ and similar terms [24]. Indeed, we might call the hedge the official flower of epidemiology. To a policy maker, journalist or member of the public not familiar with the traditions of scientific writing, the caveats and reservations may sound like the new results are irredeemably tentative, leaving us with no justification for any intervention. To those with a vested interest, the soft wording can be exploited through selective quotation and by emphasizing real or alleged weaknesses [25]. This tendency goes beyond one’s own writings and affects peer review and evaluations of manuscripts and applications. Although skepticism is in the nature of science, a malignant form is the one that is veiled and expressed in terms of need for further replication or emphasizing limitations of otherwise stable observations [9]. By softening the conclusions and avoiding attribution of specific causality and the possible policy implications, researchers protect themselves against critique by appearing well-balanced, unassuming, or even skeptical toward one’s own findings. In seeking consensus, researchers often moderate or underestimate their findings, a tendency that is not in accordance with public health interests.

These are difficult issues, requiring a balancing act. The Editors continue to ponder the question how to inspire, improve and support the best research and its translation. We believe Broadbent’s stability idea is worth considering as an alternative perspective to the replication and research translation paradigms prevalent in discussions of this topic. We also believe in Oreskes’ vision of consensus, though not to a degree that will preclude new interpretations. Meanwhile, we will endeavor to keep the Journal’s standards high while encouraging work that will make a difference.

## Data Availability

N/A
